# The fat anchor orchiopexy technique: results and outcomes from 150 cases surgical experience

**DOI:** 10.1007/s00383-021-04919-w

**Published:** 2021-05-11

**Authors:** Claudio Spinelli, Alessia Bertocchini, Gianmartin Cito, Marco Ghionzoli, Silvia Strambi

**Affiliations:** 1 Pediatric and Adolescent Surgery Division, Department of Surgical Pathology, Medical, Molecular and Critic Area, University of Pisa, Pisa, Italy; 2 Department of Urology, Careggi Hospital, University of Florence, Largo Brambilla, 3, 50134 Florence, Italy

**Keywords:** Children, Cryptorchidism, Trans-scrotal orchiopexy, Fat anchor orchiopexy

## Abstract

**Purpose:**

The purpose of the study is to evaluate results and outcomes in a long-time follow-up period, by performing a novel testicular fixation procedure, known as “fat anchor orchidopexy” (FAO), for the treatment of palpable low inguinal undescended testis.

**Materials and methods:**

We retrospectively reviewed all patients who underwent scrotal orchiopexy technique, from May 2013 to May 2019, at the Pediatric Surgery Division of Department of Surgical Pathology, University of Pisa (Italy). FAO (Spinelli’s technique) consists in anchoring the testicles to sub-scrotal fat with a single trans-scrotal incision. All the patients enrolled had history of unilateral or bilateral undescended testis. Data collected included patient’s age, operative times and complications.

**Results:**

A total of 150 children with cryptorchidism were treated using a single trans-scrotal orchiopexy. Of them, 130 patients (86.7%) had unilateral undescended testis and 20 (13.3%) bilateral cryptorchidism. Mean patient’s age was 21 months (range: 14–28 months). All the procedures were planned in a day-surgery setting. Trans-scrotal orchiopexy was successful in all cases and no patients required an additional groin incision. No intraoperatively and postoperatively major complications were observed. Patients’ post-operative pain was mild (mean pediatric visual analog scale = 2). In all cases, the healing process was rapid and no surgical wounds infections were reported during the post-operative period, referring excellent cosmesis results. During a mean 48-month follow-up period, no testicular retraction, recurrence or testis atrophy was reported.

**Conclusion:**

The original Spinelli’s technique (FAO) proves to be a safe and effective method for the treatment of palpable or distal-to-external-inguinal-ring testes. No immediate and delayed post-surgery complications were reported. In all cases, the anchored testicle remained in the scrotal position with normal vascularization. This novel surgical technique could give better options for scrotal fixation in case of low-lying cryptorchid testes.

## Introduction

Undescended testis (UDT), is defined as a failure of a single testis or both, to descend into a scrotal position [1]. Most cases of UDT are unilateral (UT), as well as from 10 to 20% both testes are involved (bilateral, BT) [2, 3]. Its incidence varies between 3 and 5% in full-term newborn babies, depending on the geographical region, ethnic group and socioeconomic status [4–6]. UDT is associated with abnormal testicular development and semen motility, and to an incorrect morphology, leading to hypotrophy and long-term infertility issues [7, 8]. It is recommended to perform orchidopexy during early childhood to prevent infertility in adulthood [9–15]. Hormone therapy, as complementary to surgical treatment, may improve sperm maturation and later semen parameters in boys with UDT [16, 17]. Depending on the position of the cryptorchid testis, the most used surgical techniques are the Shoemaker’s technique, which provides trans-inguinal access [18], Bianchi and Squire’s technique, which instead implies a trans-scrotal access [19], and his modification [20]. Children have a favorable anatomical condition to perform a less invasive approach, due to the short distance from external to the internal inguinal ring, as well as the very movable and thinner skin and subcutaneous tissue [21, 22]. These aspects suggest that a single scrotal incision, rather than a traditional inguinal incision, may be valuable for children with low palpable testes [23, 24]. This type of incision is a minimal-access approach that requires less dissection, less discomfort for the patient, and provides rapid healing, excellent cosmetic results and a good success rate [25, 26].

The purpose of the study is to evaluate results and outcomes in a long-time follow-up period, by performing a novel testicular fixation procedure, known as “fat anchor orchidopexy” (FAO) or “Spinelli’s technique”, for the treatment of palpable low inguinal undescended testis.

## Materials and methods

### Study population

We performed a retrospective analysis of 150 children with cryptorchidism, who underwent the “fat anchor orchiopexy” from May 2013 to May 2019 at the Pediatric Surgery Division of Department of Surgical Pathology, University of Pisa (Italy). All the patients enrolled had history of unilateral or bilateral undescended testis. Data collected included patient’s age, operative times and complications.

On clinical and ultrasound examination, all selected patients presented low palpable testicles. None of the patients were diagnosed with retractile testis. The a-priori exclusion criteria were: previous hormonal treatment, presence of hernia or hydrocele.

### Technique

**Fig. 1:**
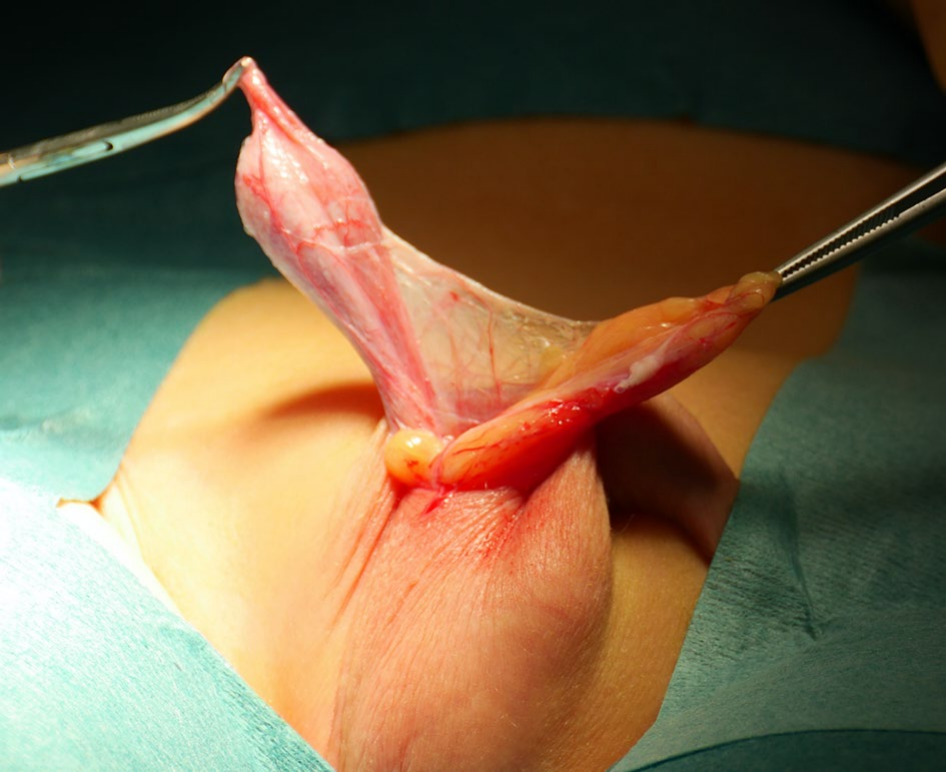
Undescended testis and pre-scrotal fat

**Fig. 2:**
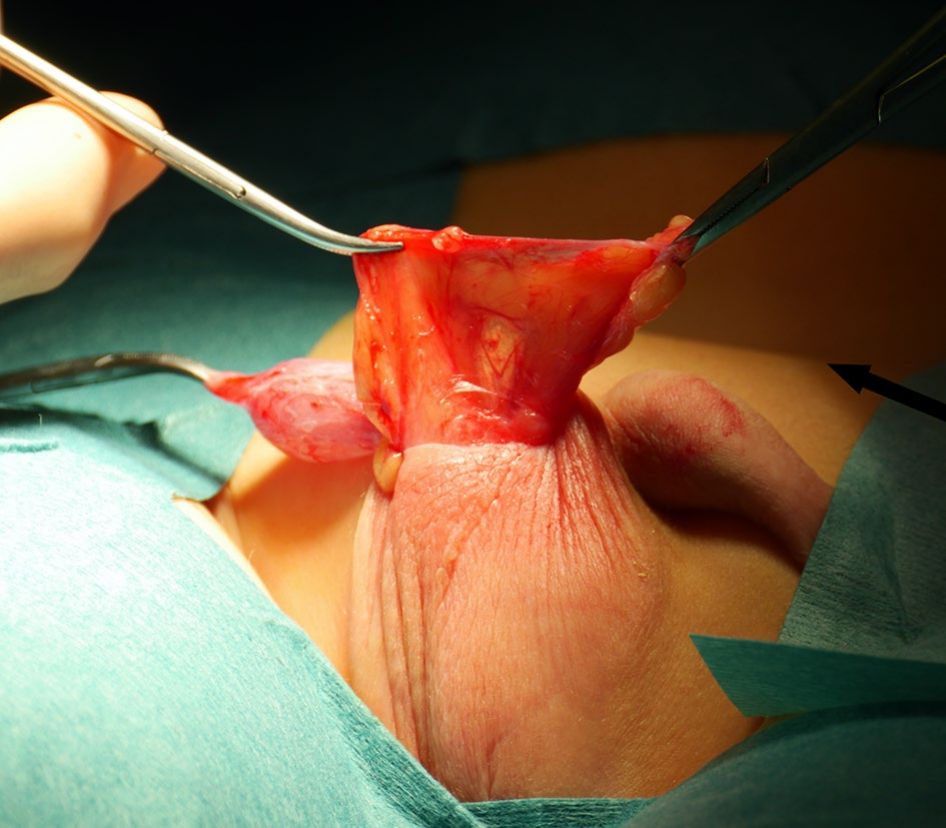
The preparation of “Fat Fan” (arrow)

**Fig. 3:**
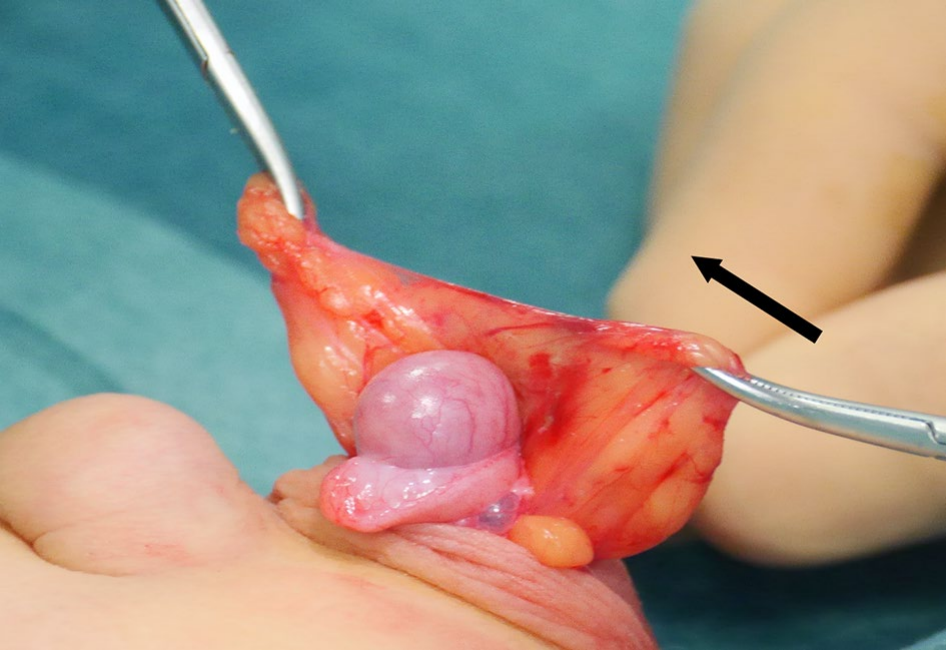
The passage of the testis through the “Fat Fan” (arrow)

**Fig. 4:**
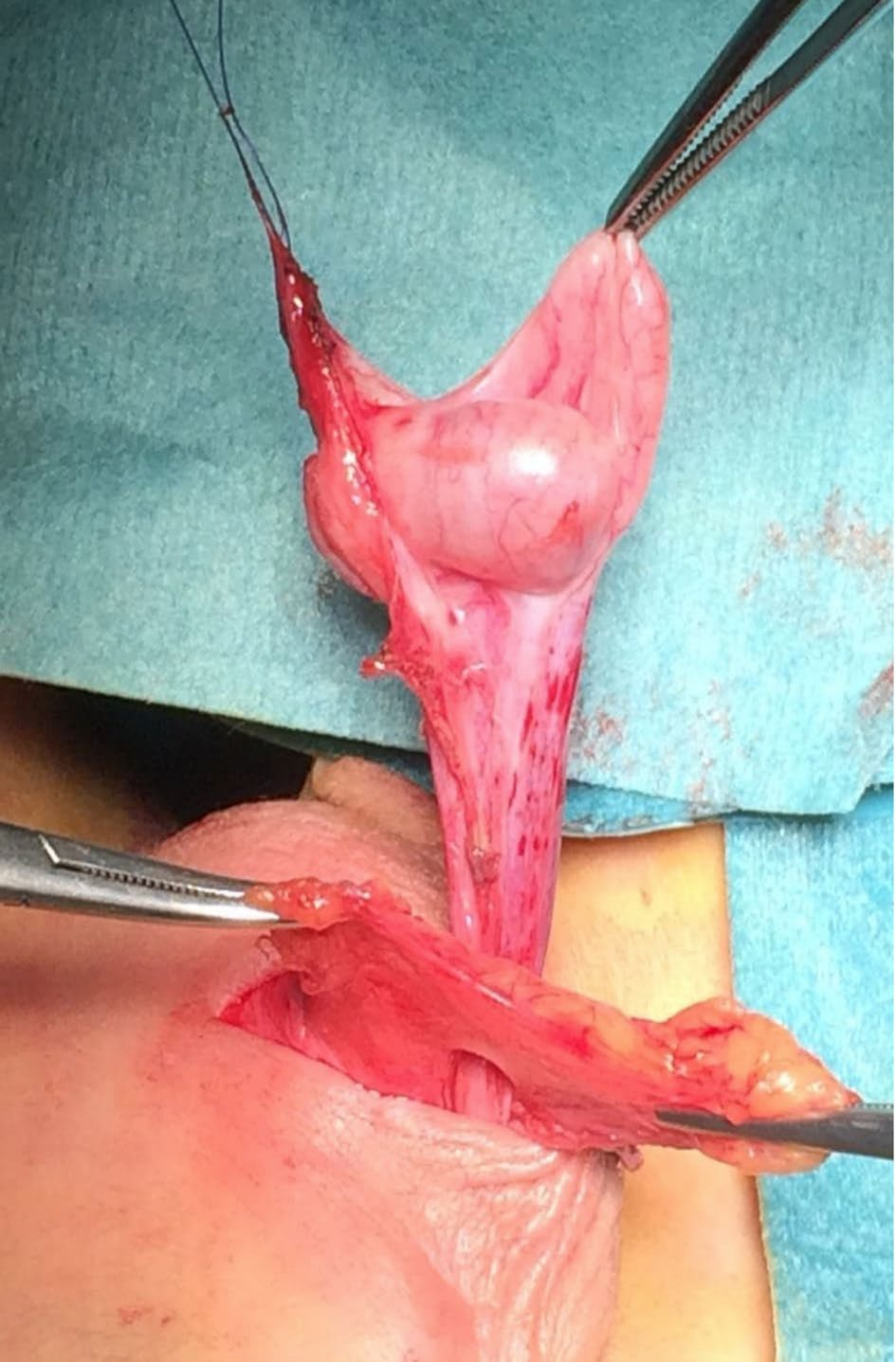
The position of the testis, after crossing of the “Fat Fan”

**Fig. 5:**
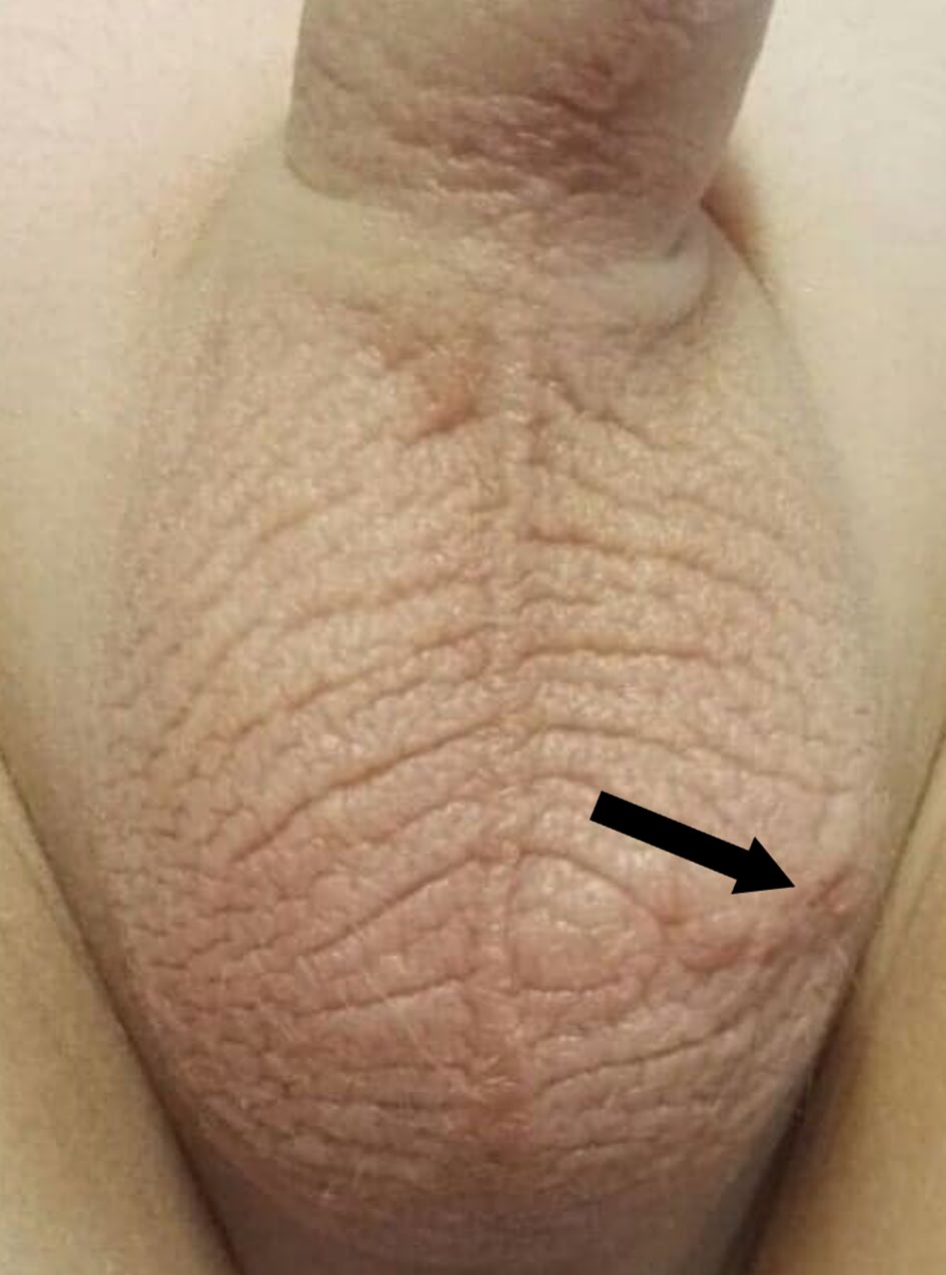
A surgical wound at 6 months from orchidopexy using “The fat anchor orchiopexy”, according to Spinelli’s technique

All the patients underwent the fat anchor orchidopexy (FAO) technique, performed by the same surgeon (C.S.) The surgical procedure was performed under general anesthesia with the patient in supine position. The actual testicular location was accurately assessed after the anesthesia induction before surgery. The testis was massaged down into the most caudal extent of the scrotum. A transverse scrotal incision was done at the level of the hemiscrotum, though which the testicle was exposed and delivered. The external spermatic fascia covering the spermatic cord was then meticulously dissected from its surrounding fat pad. Later, a fan of adipose tissue of the sub-scrotal fat of trapezoidal shape—so called “fat fan” was prepared (Figs. 1, 2). The sub-scrotal fat represents a continuation of the superficial fascia (Camper’s fascia and Scarpa’s fascia) covering the anterior abdominal wall [27–29]. This pad of fat is well represented in small children, but tends to decrease as age increases. An accurate funiculolisis was carried out until reaching the external inguinal ring. The external spermatic fascia, cremasteric muscle and internal spermatic fascia were incised and separated from the vas deferens and spermatic vessel. Thereafter, the vaginal tunic of the testis was opened and the processus vaginalis was possibly examined, dissected free and ligated high, as would be done for conventional herniotomy. Once the testis was mobilized, a keyhole was performed through the mid portion of the “fat-fan” and the testis is passed through it (Figs. 3, 4). Subsequently, the testis was anchored to the adipose fan with two points, one medial and one lateral (using 4/0 mono thread absorbable), to prevent testicular ascending. Thus, the testis was placed inside the ipsilateral scrotum without tension. Finally, dartos and scrotal skin were sutured 5/0 absorbable stitches (Fig. 5).

### Main outcomes measures

Postoperative pain was assessed using the pediatric visual analog scale (VAS). The VAS consisted of a 100-mm horizontal line without any other visual markers on or around it [30]. Intraoperative and post-operative complications were recorded. Each patient underwent a clinical examination and abdominal/scrotal-groin ultrasounds at 3 months, 6 months and 12 months after surgery and then annually. Additionally, a monthly self-examination of the testicles was suggested to the patients, as advised by Radmayr [31].

All procedures performed were in accordance with the ethical standards of the institutional and/or national research committee and with the 1975 Helsinki Declaration.

## Results

The fat anchor orchiopexy technique was performed in 150 patients: 130 (86.7%) presented UT-UDT, 20 (13.3%) had BT-UDT. The mean patient’s age at surgery was 21 months (range: 14–28 months). All the procedures were planned in a day-surgery setting. The mean operating time was 37 min (range: 30–50 min) in case of UT-UDT. In 60 patients (40.0%), the patency of the processus vaginalis was detected, requiring a careful dissection with high ligature. In all cases, no intraoperative complications occurred. Patient’s post-operative pain was mild (mean visual analog scale = 2). All patients received a pain regimen with acetaminophen as needed until the first post-operative day, not requiring additional narcotic pain medication. Twenty patients (13.3%) needed antalgic therapy up to 3 days after surgery. No hormonal therapy was given postoperatively. No post-operative complications were recorded. All the children returned to their normal activities 3 days after surgery. In all cases, the healing process was rapid and no surgical wounds infections were reported during the post-operative period, referring excellent cosmesis results.

A mean follow-up period of 48 months (range: 18—84 months) was performed. No testicular retraction, recurrence or testis atrophy was reported.

## Discussion

The principles which inspire the surgical treatment of UDT, described by Park and Choi [32], according to Bevan’s studies [33] are: the mobilization of the testis, spermatic vessels and deferent, the repair of associated hernia or better the peritoneo-vaginal duct, and the testicular fixation in the scrotal bag. Many surgical techniques have been proposed to anchor the testicle, after it was brought down into the scrotum, to maintain its position; however, the optimal method remains controversial. Ombredanne et al. in 1945 [34] first introduced fixing the testis into the contralateral scrotal pouch through a window in the scrotal septum. Cabot and Nesbit in 1931 [35] anchored the testes to the contralateral thigh using a rubber band attached to a silk suture. Also Torek et al. in 1931 [36] proposed to anchor the testis to the fascia of the thigh. Shoemaker in 1932 [18] and later Lattimer in 1957 [37], suggested fixing the testes in a subcutaneous position, in an extradartos pouch, between dartos and scrotal skin. The incorporation of the sub-scrotal fat in fixing the testes inside the scrotum was first suggested by Spinelli et al. in 2017 [27, 38]. The surgical approach depends on the testicular position on physical examination. Most of the orchidopexies for palpable testicles are performed through an inguinal incision, although a scrotal approach can be safely used depending on the position of the testis [39, 40]. The possibility of a scrotal approach is allowed by the fact that, in the majority of palpable undescended testicles, the testicular vessels and the vasa, after dissection of the spermatic fascia, cremasteric muscle and the processus vaginalis, are long enough to allow the testes to reach the scrotum without tension, as confirmed by the experiences of Bianchi and Squire [19] and Hazebroek et al. [41]. Both inguinal and scrotal orchiopexy are two traditional approaches with high efficacy, both performed as relatively quick and without complications [42]. This issue is confirmed by a meta-analysis performed by Feng et al. [43] and a study conducted by Al-Mandil’s et al. [44], which highlight how the trans-scrotal orchiopexy is associated with shorter operating times when compared with the standard inguinal orchidopexy. According to literature [45], the duration of the scrotal approach ranges from 18.9 to 40.5 min, making this procedure significantly shorter than the inguinal approach, as shown in our study. In addition, Hyuga’s et al. [45] demonstratede a slightly lower incidence of post-operative wound infection in the trans-scrotal approach, compared to the inguinal one (1.1 vs. 2.5%, respectively), although this difference is not significant. According to other authors [46–48], the single scrotal incision has the advantages of lower post-operative pain, compared with the inguinal access.

Moreover, Novaes et al. [49] reported, after a single scrotal-incision orchidopexy: 1.43% of relapse, 0.1% of persistent or recurrent hernia and 0.3% of testicular atrophy, resulted by sperm vessels injury during surgery or even to the spermatic cord itself. However, in our case series, all the patients did not report complications, during a long-term follow-up period, confirming the scrotal position of the testes.

## Conclusions

The original Spinelli’s technique provides a trans-scrotal access, using a fan of adipose tissue. This approach proves to be a safe and alternative method to traditional inguinal approach for palpable or distal-to-external-inguinal-ring testes, ensuring excellent cosmetic results. Moreover, this technique is associated with a shorter operating time, not reporting immediate and delayed post-surgery complications. This novel surgical technique could give better options for scrotal fixation in case of low-lying cryptorchid testes.

## Abbreviations

UDT: Undescended testis
UT-UDT: Unilateral undescended testis
BT-UDT: Bilateral undescended testis
FAO: Fat anchor orchiopexy
